# Editorial: Reviews in cellular neurophysiology 2022: neurophysiological mechanisms in the aging brain

**DOI:** 10.3389/fncel.2024.1385783

**Published:** 2024-02-29

**Authors:** Joshua L. Plotkin, Steven M. Graves, Markus Riessland

**Affiliations:** ^1^Department of Neurobiology and Behavior, Center for Nervous System Disorders, Stony Brook University, Stony Brook, NY, United States; ^2^Department of Pharmacology, University of Minnesota, Minneapolis, MN, United States

**Keywords:** aging, inflammaging, neurophysiological mechanism, brain, neurodegeneration

The aging process of the human brain is deserving of significant attention due to the fact that aging is a primary risk factor for numerous neurodegenerative disorders (Hou et al., 2019). Intriguingly, despite this, much remains unclear about the neurophysiological changes that occur during the aging process. Research into age-related neurodegeneration has highlighted inflammation-driven aging, commonly referred to as “inflammaging,” as a central mechanism underlying many neurodegenerative disorders (Franceschi et al., 2007). The excessive activation of immune and non-immune cells in the brain, such as microglia and astrocytes, appears to substantially contribute to neuronal loss with advancing age (Deleidi et al., 2015). Neuroinflammation, stemming from various mechanisms, emerges as a pivotal factor in numerous diseases, as well as age-related neurodegeneration more broadly. This mechanism could contribute to the development of age-related ailments such as Alzheimer's disease (AD), Parkinson's disease (PD), amyotrophic lateral sclerosis (ALS), and others (Russo and Riessland, 2022). Unraveling the underlying mechanisms of general neuroinflammation and neurophysiological changes in the aging brain could hold the key for the identification and rational development of neuroprotective therapies that could prove to be beneficial in a multitude of age-related neurodegenerative diseases.

One approach to assess neuroinflammation was reviewed by Karvandi et al.. The authors provide a broad perspective on shared mechanisms underlying neuronal loss in neurodegenerative diseases during aging. The authors explore the interconnected pathways leading to neuronal cell death, with a focus on endoplasmic reticulum (ER) stress, oxidative stress, and neuroinflammation. This overview sets the stage for understanding the aging-related intricacies of neuronal loss and suggests potential neuroprotective strategies. The authors note that mechanisms such as protein misfolding and aggregation, mitochondrial dysfunction, generation of reactive oxygen species (ROS), and activation of the innate immune response are the most critical hallmarks of common neurodegenerative diseases. Consequently, they review ER stress, oxidative stress, and neuroinflammation as major pathological factors of neuronal cell death and discuss the neuroprotective effects of approaches that target these pathways.

In line with the observation that molecular stress can cause neurological diseases is a contribution describing the function of cyclin-dependent kinase 5 (Cdk5). As reviewed by Ao et al., Cdk5 is critical for the development of the nervous system, the migration and differentiation of neurons, the formation of synapses, and axon regeneration. The authors comprehensively review the involvement of Cdk5 in several age-related neurological diseases such as AD, PD, ALS, multiple sclerosis (MS) and others. Their review sheds light on the role of Cdk5 during neuronal development and in the aging nervous system. Highlighting the role of Cdk5 in various neurological disorders and its potential as a therapeutic target, the article underscores the importance of understanding its associated physiological and pathological mechanisms in the aging brain. This exploration into Cdk5 offers valuable insights into aging-related aberrations and potential avenues for targeted treatments. Finally, Ao's outlook of the potential therapeutic applicability of Cdk5 inhibitors seems promising, while the need for the development of more selective inhibitors is brought to attention.

A third comprehensive review in this Research Topic collection provided by Croucher and Fleming aligns seamlessly with the focus on stress-induced changes in neurophysiology, exploring the intricate role of ATP13A2 in various neurodegenerative conditions, including PD, Kufor-Rakeb Syndrome, neuronal ceroid lipofuscinosis, hereditary spastic paraplegia, and ALS. The article discusses how ATP13A2 mutations may interact with environmental exposures, emphasizing the relevance of gene-environment interactions in the brain, particularly the vulnerable basal ganglia. In line with this, the authors discuss that heavy metal toxicity including manganese, iron, and zinc has been connected to ATP13A2 dysfunction. By unraveling common pathological mechanisms, the review contributes to a deeper understanding of ATP13A2-related disorders and their implications for impairment of cellular neurophysiology.

Finally, the research article by Yu et al. aims to understand the role of Piezo1, a mechanosensitive ion channel, in astrocytes of the mouse cerebellum. Through a battery of experiments involving electrophysiological recordings, calcium imaging, and cell migration assays, the study investigates the impact of lipopolysaccharide (LPS)-induced neuroinflammatory conditions on astrocytic Piezo1 currents. Using the above techniques and pharmacological tools (such as the Piezo1 agonist Yoda1 and inhibitor GsMTx4), the authors report that LPS treatment sensitizes Piezo1 channels in mouse astrocytes. These findings not only shed light on the LPS-sensitizing effect on Piezo1 channels in astrocytes but also provide potential implications for neuroinflammation pathogenesis. Yu et al. propose that astrocytic Piezo1 plays a critical role in the pathogenesis of neuroinflammation, potentially serving as a cornerstone for future research aimed at alleviating inflammation-mediated neurodegeneration.

Overall, the four independent articles in this special issue not only highlight neurophysiological mechanisms in the aging brain from diverse angles, but also show a clear convergence on commonalties among diverse neurodegenerative diseases and molecular pathways.

## Author contributions

JP: Writing – original draft, Writing – review & editing. SG: Writing – original draft, Writing – review & editing. MR: Conceptualization, Writing – original draft, Writing – review & editing.

## References

[B1] Deleidi M. Jäggle M. Rubino G. (2015). Immune aging, dysmetabolism, and inflammation in neurological diseases. Front Neurosci. 9:172. 10.3389/fnins.2015.00172 26089771 PMC4453474

[B2] Franceschi C. Capri M. Monti D. Giunta S. Olivieri F. Sevini F.. (2007). Inflammaging and anti-inflammaging: a systemic perspective on aging and longevity emerged from studies in humans. Mech Ageing Dev. 128:92–105. 10.1016/j.mad.2006.11.016 17116321

[B3] Hou Y. Dan X. Babbar M. Wei Y. Hasselbalch S. G. Croteau D. L.. (2019). Ageing as a risk factor for neurodegenerative disease. Nat Rev Neurol. 15:565–581. 10.1038/s41582-019-0244-7 31501588

[B4] Russo T. Riessland M. (2022). Age-Related Midbrain Inflammation and Senescence in Parkinson's Disease. Front Aging Neurosci. 14:917797. 10.3389/fnagi.2022.917797 35721008 PMC9204626

